# A novel bHLH transcription factor PlLHL1 targets *PlLAC15* to negatively regulate stem strength by inhibiting syringyl lignin deposition in herbaceous peony

**DOI:** 10.1093/hr/uhaf080

**Published:** 2025-03-10

**Authors:** Ziyi Liu, Yuehan Yin, Shiqi Zuo, Minghao Zhao, Jun Tao, Yuhan Tang, Daqiu Zhao

**Affiliations:** College of Horticulture and Landscape Architecture, Yangzhou University, Wenhui East Road 48, Yangzhou, Jiangsu 225009, China; College of Horticulture and Landscape Architecture, Yangzhou University, Wenhui East Road 48, Yangzhou, Jiangsu 225009, China; College of Horticulture and Landscape Architecture, Yangzhou University, Wenhui East Road 48, Yangzhou, Jiangsu 225009, China; College of Horticulture and Landscape Architecture, Yangzhou University, Wenhui East Road 48, Yangzhou, Jiangsu 225009, China; College of Horticulture and Landscape Architecture, Yangzhou University, Wenhui East Road 48, Yangzhou, Jiangsu 225009, China; College of Horticulture and Landscape Architecture, Yangzhou University, Wenhui East Road 48, Yangzhou, Jiangsu 225009, China; College of Horticulture and Landscape Architecture, Yangzhou University, Wenhui East Road 48, Yangzhou, Jiangsu 225009, China

Dear Editor,

Herbaceous peony (*Paeonia lactiflora* Pall.) is a newly emerging high-end cut flower in recent years, and the stem bending caused by insufficient stem strength greatly affects its commercial value. Our previous studies have confirmed that lignin could serve as a mechanical support for *P. lactiflora* stems, and the syringyl lignin (S-lignin) had a significant impact on its stem strength [1]. Meanwhile, a series of lignin biosynthetic genes have been isolated in *P. lactiflora*, and their roles in lignin accumulation of stems were validated using *P. lactiflora* virus-induced gene silencing (VIGS) technology and tobacco overexpression technology [2–4]. However, the studies on transcriptional regulatory networks of lignin biosynthetic genes have been limited in *P. lactiflora*.

Recently, the laccase gene (*PlLAC15*) has been reported to enhance *P. lactiflora* stem strength through promoting the accumulation of S-lignin [5]. To explore the transcriptional regulatory network, its upstream transcriptional regulatory factors were screened by the yeast one-hybrid (Y1H) system. The transcription factor (TF) bHLH155-like was obtained and its full length of 2037 bp was cloned, which encoded 678 amino acids. Based on the phylogenetic tree constructed from its protein sequence and 171 *Arabidopsis* bHLHs, it was named PlLHL1 with the greatest degree of protein sequence similarity with AtbHLH173/LHL1. Sequence alignment of PlLHL1, AtbHLH173/AtLHL1, and AtbHLH155/AtLHL2 showed that PlLHL1 contained a highly conserved bHLH domain (https://doi.org/10.6084/m9.figshare.28427126.v1).

bHLH TFs modulate the expression levels of target genes by binding to the motifs in their promoters, either E-box (CANNTG) or G-box (CACGTG). Among the upstream 2000 bp promoter fragment of *PlLAC15*, a total of eight E-box motifs were identified (Fig. 1A). Subsequently, Y1H validation confirmed that PlLHL1 physically bound to the promoter of *PlLAC15* (Fig. 1B). Dual-luciferase reporter (DLR) assay revealed that PlLHL1 exerted a notable transcriptional repressive effect on the promoter activity of *PlLAC15* (Fig. 1C). Furthermore, the electrophoretic mobility shift assay (EMSA) experiment confirmed the binding of PlLHL1 to the E-box motif within the promoter of PlLAC15 (Fig. 1D). These results suggested that PlLHL1 could repress the expression of *PlLAC15*.

**Figure 1:**
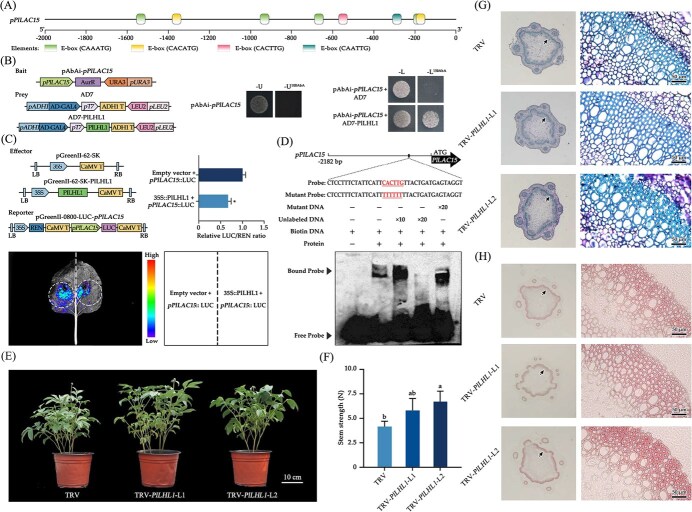
PlLHL1 binds to the promoter of *PlLAC15* and regulates S-lignin deposition. (A) Analysis of E-box elements within the promoter region of *PlLAC15*. (B) Interaction identification between PlLHL1 and the promoter of *PlLAC15* based on Y1H. (C) DLR assay to identify the interaction between PlLHL1 and the promoter of *PlLAC15*. (D) EMSA assay to identify the interaction between PlLHL1 and the promoter of *PlLAC15.* (E) Effects of silencing *PlLHL1* on phenotype. (F) Effects of silencing *PlLHL1* on stem strength. (G) Transverse sections of stems of the *PlLHL1*-silenced plants stained with toluidine blue method. (H) Transverse sections of stems of the *PlLHL1*-silenced plants stained with maüle method. Statistical analyses were conducted using Duncan’s test, and significant differences (*P* < 0.05) denoted by ‘*’ and letters.

To determine whether PlLHL1 plays the function of TF, we firstly observed its subcellular localization, and found that PlLHL1 localized to the nucleus. Subsequently, we investigated the expression pattern of *PlLHL1*. The highest expression level of *PlLHL1* was found in stems and the lowest in roots. And the expression level of *PlLHL1* in vascular tissues gradually decreased with stem development, with the highest levels at both P1 and P2 periods and the lowest at P4 period (https://doi.org/10.6084/m9.figshare.28427126.v1). These suggested PlLHL1 was a nuclear-localized TF, which might be associated with stem development.

Moreover, *PlLHL1* was transiently silenced by tobacco rattle virus (TRV)-mediated VIGS in *P. lactiflora* (Fig. 1E). The *PlLHL1*-silenced plants were identified, and the silencing efficiency of *PlLHL1* was 21% and 61% compared to the control plants, and *PlLAC15* was significantly expressed in the *PlLHL1*-silenced plants. When compared with control plants, plant height, stem diameter, and stem weight showed no significant changes (https://doi.org/10.6084/m9.figshare.28427126.v1), whereas stem strength significantly increased in the *PlLHL1*-silenced plants (Fig. 1F).

To further investigate the effect of silencing *PlLHL1* on xylem development and lignin accumulation of *P. lactiflora* stems, the stem cross-sections from *PlLHL1*-silenced plants and the control plants were stained with toluidine blue. Compared with the control plants, the width of the xylem and the intensity of blue staining were significantly higher, while the cell layers of the xylem showed no significant changes in the *PlLHL1*-silenced plants (Fig. 1G), this result was in accordance with the statistical data and lignin content (https://doi.org/10.6084/m9.figshare.28427126.v1). Furthermore, the stem cross-sections were stained with maüle method to observe S-lignin deposition. The intensity of red staining of the xylem in the *PlLHL1*-silenced stems was higher than that in the control (Fig. 1H), suggesting that more S-lignin was deposited in the xylem of *PlLHL1*-silenced stems.

Collectively, these data supported that PlLHL1 targeted *PlLAC15* to negatively regulate stem strength by inhibiting S-lignin deposition in *P. lactiflora*. These finds will improve our comprehension of the molecular mechanism by which the S-lignin deposition regulates stem strength in plants.

## Data Availability

Data are contained within the article or Supplementary Data files. The following supporting information can be downloaded at: https://doi.org/10.6084/m9.figshare.28427126.v1
